# Assessing brain microstructural changes in chronic kidney disease: a diffusion imaging study using multiple models

**DOI:** 10.3389/fneur.2024.1387021

**Published:** 2024-05-01

**Authors:** Limei Han, Jie Yang, Chao Yuan, Wei Zhang, Yantao Huang, Lingli Zeng, Jianquan Zhong

**Affiliations:** ^1^Department of Radiology, Affiliated Hospital of North Sichuan Medical College, Nanchong, Sichuan Province, China; ^2^Department of Radiology, Zigong First People's Hospital, Zigong, Sichuan Province, China

**Keywords:** chronic kidney disease, brain microstructure, diffusion tensor imaging, mean apparent propagator magnetic resonance imaging, neurite orientation dispersion and density imaging

## Abstract

**Objectives:**

To explore the effectiveness of diffusion quantitative parameters derived from advanced diffusion models in detecting brain microstructural changes in patients with chronic kidney disease (CKD).

**Methods:**

The study comprised 44 CKD patients (eGFR<59 mL/min/1.73 m^2^) and 35 age-and sex-matched healthy controls. All patients underwent diffusion spectrum imaging (DSI) and conventional magnetic resonance imaging. Reconstructed to obtain diffusion MRI models, including diffusion tensor imaging (DTI), neurite orientation dispersion and density imaging (NODDI) and Mean Apparent Propagator (MAP)-MRI, were processed to obtain multi-parameter maps. The Tract-Based Spatial Statistics (TBSS) analysis was utilized for detecting microstructural differences and Pearson correlation analysis assessed the relationship between renal metabolism markers and diffusion parameters in the brain regions of CKD patients. Receiver operating characteristic (ROC) curve analysis assessed the diagnostic performance of diffusion models, with AUC comparisons made using DeLong’s method.

**Results:**

Significant differences were noted in DTI, NODDI, and MAP-MRI parameters between CKD patients and controls (*p* < 0.05). DTI indicated a decrease in Fractional Anisotropy(FA) and an increase in Mean and Radial Diffusivity (MD and RD) in CKD patients. NODDI indicated decreased Intracellular and increased Extracellular Volume Fractions (ICVF and ECVF). MAP-MRI identified extensive microstructural changes, with elevated Mean Squared Displacement (MSD) and Q-space Inverse Variance (QIV) values, and reduced Non-Gaussianity (NG), Axial Non-Gaussianity (NGAx), Radial Non-Gaussianity (NGRad), Return-to-Origin Probability (RTOP), Return-to-Axis Probability (RTAP), and Return-to-Plane Probability (RTPP). There was a moderate correlation between serum uric acid (SUA) and diffusion parameters in six brain regions (*p* < 0.05). ROC analysis showed the AUC values of DTI_FA ranged from 0.70 to 0.793. MAP_NGAx in the Retrolenticular part of the internal capsule R reported a high AUC value of 0.843 (*p* < 0.05), which was not significantly different from other diffusion parameters (*p* > 0.05).

**Conclusion:**

The advanced diffusion models (DTI, NODDI, and MAP-MRI) are promising for detecting brain microstructural changes in CKD patients, offering significant insights into CKD-affected brain areas.

## 1 Introduction

Chronic kidney disease (CKD) is a long-term condition marked by renal damage or a reduced glomerular filtration rate (eGFR) under 60 mL/min/1.73m^2^ for over 3 months. With a global prevalence of about 9.1%, CKD presents a major public health issue (1). CKD increases the risk of cerebrovascular diseases (2, 3) and leads to oxidative stress escalation and vascular integrity compromise, resulting in neurological decline and structural brain damage, including cognitive impairment, brain atrophy, cerebral microhemorrhage and demyelination changes (4–6). Consequently, early detection of neuroimaging changes in CKD patients is vital for effective treatment and prognosis.

Magnetic resonance imaging (MRI) is a non-invasive technique extensively employed in clinical settings to identify structure and function changes in the brain. Studies on diffusion tensor imaging (DTI) have confirmed that the integrity of brain white matter in patients with CKD is altered (7–9). Among others, Liu et al. also pointed out the existence of an association between abnormal WM integrity and clinical indicators (such as hemoglobin, serum urea, serum creatinine, serum calcium, and serum potassium levels).

However, DTI has certain limitations when analyzing nerve fiber intersection areas. Recently, more advanced models have been developed, such as neurite oriented diffusion and density imaging (NODDI) and mean apparent spread volume (MAP-MRI), able to provide clinically more specific characterization about the internal and external environment of nerve cells and the configuration of nerve fibers. Notably, the human body comprises different tissue types, each characterized by distinct dispersion sizes and directions. NODDI, enhances the understanding of the microstructural features of tissues by transcending the limitations of traditional DTI and non-Gaussian model-based approaches, categorizing brain tissue into three different compartments: intracellular volume fraction (ICVF), extracellular volume fraction (ECVF), and cerebrospinal fluid (10). In contrast to DTI and NODDI, MAP-MRI represents a cutting-edge, advanced quantitative diffusion model. MAP-MRI encompasses several key parameters, including return-to-origin probability (RTOP), return-to-axis probability (RTAP), return-to-plane probability (RTPP), mean squared displacement (MSD), q-space inverse variance (QIV) and non-Gaussianity (NG). These parameters are sensitive to diffusion restriction and tissue composition variations (11).

Currently, NODDI and MAP-MRI have been applied in studying central nervous system diseases such as Alzheimer’s disease (12–15) and Parkinson’s disease (16–19), showing more advantages than DTI in describing complex nerve fiber structures in some studies. Research also extends to the identification, grading, and evaluation of central nervous system tumors (20, 21). Some researchers have asserted that NODDI and MAP-MRI represent formidable tools for *in vivo* assessment of brain microstructure, and they can provide new insights into the intricacies of cerebral tissue (10, 11). Nevertheless, to our knowledge, there have been limited reports on the application of NODDI and MAP-MRI models in evaluating both the microstructure and function of the brain in CKD patients (22). Moreover, there is a scarcity of studies comparing the diagnostic efficacy of DTI, NODDI and MAP in detecting changes in brain microstructure among CKD patients. Therefore, we intend to do a preliminary exploration of using diffusion MRI models to detect brain microstructure information among individuals with CKD.

Additionally, these diffusion MRI models can be simultaneously derived from diffusion spectral imaging (DSI), a grid acquisition model that reconstructs various diffusion MRI models through the raw diffusion data. This method not only epitomizes efficiency and cost-effectiveness but also facilitates the comparative evaluation of different models in discerning the brain’s microstructural nuances.

However, this study did not include diffusion kurtosis imaging (DKI), instead DTI, NODDI, and MAP-MRI were chosen because of their proven track record, clinical applicability, and the distinct but complementary information they provide about brain tissue microstructure. From general diffusion features (DTI) and neurite structure (NODDI) to complex water molecular diffusion patterns (MAP-MRI), this selection fits into our initial exploration of the expected microscopic changes in the brain in patients with CKD. Moreover, the inclusion of DKI, would require additional validation and analysis, potentially complicating the interpretation of our primary endpoints without significantly enhancing our understanding of CKD-related changes. However, we acknowledge the utility of DKI in broader neuroimaging research and suggest that future studies could include DKI to provide a more comprehensive assessment of microstructural alterations in CKD.

In this study, we conduct a preliminary prospective analysis using the DSI data,16 quantitative microstructural parameters derived from DTI, NODDI, MAP-MRI diffusion models to explore the changes in brain microstructure in CKD patients, and evaluate the correlation of these diffusion parameters with clinical experimental indicators related to renal function in CKD patients. Ultimately, we aimed to evaluate the diagnostic value of these parameters in reflecting CKD-related brain alterations.

## 2 Materials and methods

### 2.1 Participants

This study received approval from the Ethics Committee, and all subjects signed informed consent forms. A total of 44 CKD patients diagnosed were recruited in the study. The exclusion criteria were as follows: (1) obvious neurological disease, mental disease; (2) Traumatic brain injury, cerebral infarction, brain tumor; (3) Magnetic resonance imaging (MRI) to examine contraindications; (4) Audio-visual impairment, (5) poor-quality MR images due to movement artifacts. The inclusion criteria were as follows: (1) CKD patients diagnosed in the Department of Nephrology of our hospital followed the kidney disease outcomes quality initiative (K/DOQI) criteria with eGFR<59 mL/min/1.73 m^2^; (2) No history of kidney transplantation; (3) No drug abuse. In addition, 35 healthy volunteers, matched for sex and age, were recruited in this study.

### 2.2 Data acquisition

All subjects underwent structural MRI and DSI on a 3.0 T MRI scanner (MAGNETOM VIDA, Siemens Healthcare, Erlangen, Germany) with a 64-channel head coil. The DSI sequence was obtained in the axial plane using a half q-space Cartesian grid sampling procedure under the following parameters: TR/TE = 4400/97 ms; FOV = 220 × 220 mm^2^; SMS factor = 2; slice thickness = 2.0 mm; voxel size = 2.0× 2.0× 2.0mm^3^; number of slices = 60; a total of 18 b-values = 0, 200, 350, 400, 550, 750, 950, 1,150, 1,500, 1,700, 1,850, 1,900, 2,050, 2,100, 2,250, 2,450, 2,650, and 3,000 s/mm^2^; a total of 128 diffusion sampling were acquired. High resolution 3D T1-weighted imaging was performed using the MPRAGE sequence, with the following parameters: TR = 2,300 ms, TE = 2.45 ms, flip angle = 9°, FOV = 256 × 256 mm^2^, thickness = 0.9 mm, number of slices = 176, voxel size = 1 × 1 × 1 mm^3^, and the total scan time was 4 min 8 s.

### 2.3 Diffusion data analysis

**Figure 1:**
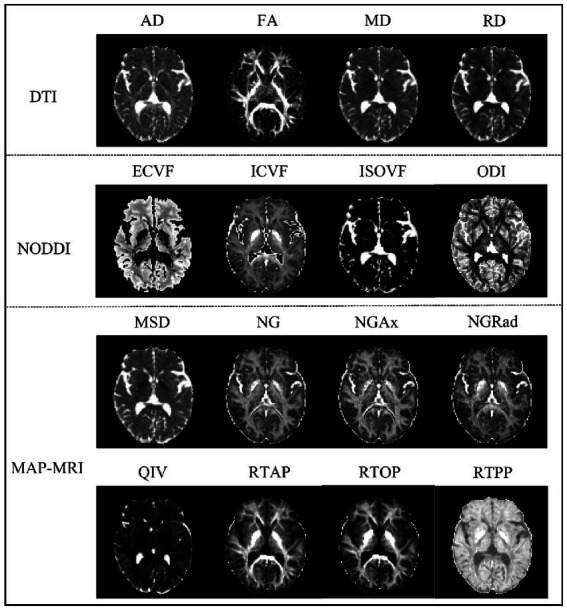
Comparison of three diffusion MRI maps showing brain microstructure. The top panel shows DTI parameters with Axial Diffusivity (AD), Fractional Anisotropy (FA), Mean Diffusivity (MD), and Radial Diffusivity (RD). The middle panel presents NODDI parameters: Extracellular Volume Fraction (ECVF), Intracellular Volume Fraction (ICVF), Isotropic Volume Fraction (ISOVF), and Orientation Dispersion Index (ODI). The bottom panel displays MAP-MRI parameters: Mean Squared Displacement (MSD), Non-Gaussianity (NG), Axial Non-Gaussianity (NGAx), Radial Non-Gaussianity (NGRad), Q-space Inverse Variance (QIV), Return-to-Axis Probability (RTAP), Return-to-Origin Probability (RTOP), and Return-to-Plane Probability (RTPP). Each parameter map reveals specific aspects of brain tissue microstructure.

Multiple parameter maps of DTI, MAP-MRI and NODDI were obtained from DSI data calculated using an in-house prototype software developed by MR Station (Chengdu Zhongying Medical Technology Co., Ltd.). All raw diffusion imaging data need to be converted to NIFTI format and subjected to eddy current correction (FSL). The maps of quantitative microstructural parameters are shown in Figure 1. The quantitative microstructural parameters are as follows:

DTI: axial diffusivity (AD), fractional anisotropy (FA), mean diffusivity (MD), Radial diffusivity (RD).

NODDI: extracellular volume fraction (ECVF), intracellular volume fraction (ICVF), isotropic volume fraction (ISOVF), orientation dispersion index (ODI).

MAP-MRI: mean squared displacement (MSD), non-Gaussianity (NG), Axial non-Gaussianity (NGAx), radial non-Gaussianity (NGRad), q-space inverse variance (QIV), return-to-axis probability (RTAP), return-to-origin probability (RTOP), return-to-plane probability (RTPP).

### 2.4 Tract-based spatial statistics processing

In order to evaluate the difference between CKD patients and healthy controls, diffusion parameter maps underwent processing through the Tract-Based Spatial Statistics (TBSS) pipeline within the FSL toolbox from the Oxford Center for Functional MRI of the Brain.^1^ This involved: (a) nonlinear registration of FA maps for CKD and healthy controls to the FMRIB58_FA template; (b) affine alignment of all subjects to the 1 × 1 × 1 mm^3^ Montreal Neurological Institute (MNI152) standard space; (c) averaging, skeletonizing, and creating a mean FA skeleton from aligned images; (d) projecting FA and other parameter maps onto this mean FA skeleton. The brain was segmented into 50 regions using Johns Hopkins University (JHU) White Matter-labels as masks.

### 2.5 Statistical analysis

For group difference analysis, nonparametric permutation tests were performed utilizing the randomize tool. The random permutations were set to 5,000. Threshold-free cluster enhancement (TFCE) was used for multiple comparison correction. The statistical threshold was set at *p* < 0.001. Finally, the results of comparison between groups were overlaid on JHU-White Matter-labels templates to localization of significant brain regions.

Statistical analyses were performed by using the IBM SPSS 23.0 (IBM Corp, Armonk, NY, United States) software package. Pearson correction analysis was used to evaluate relationship between clinical data and diffusion parameter maps. The criterion for statistical significance was *p* < 0.05, with false discovery rate (FDR) used to correct for multiple comparisons.

Receiver operating characteristic (ROC) curves were obtained and the area under the receiver operating characteristic curves (AUC) was calculated to measure the predictive performance of the diffusion MRI models in identifying structural changes in the CKD brain. The AUCs of different diffusion models were compared using Delong’s method. A significance threshold of *p* < 0.05 was applied, with differences reaching this level considered statistically significant.

## 3 Results

### 3.1 Study population

44 patients of CKD (27 males and 17 females; mean age, 61.205 ± 11.496 years; age range, 43–86 years) and 35 healthy volunteers (12 males and 23 females; mean age, 63.286 ± 11.052 years; age, 42–87 years) were included in our study. There were no significant differences observed in terms of age and gender between the two groups. The demographic data results are presented in Table 1.

**Table 1:** Characteristics of subjects.

	CKD (*n* = 44)	HC (*n* = 35)	*p-*value
Age(years)	43–86(61.205 ± 11.496)	42–87 (60.731 ± 10.654)	*p* = 0.425
Gender (Male/Female)	27/17	12/23	*p* = 0.690
Hypertension	24 (54.5%)	0	NA
Diabetes	19 (43.2%)	0	NA
Duration (years)	0–15 (3.984 ± 4.422)	0	NA

Values are expressed as mean ± standard deviation. CKD, chronic kidney disease; HC, healthy controls; NA, not applicable.

### 3.2 Diffusion quantitative parameters of DTI, NODDI and MAP-MRI

**Figure 2:**
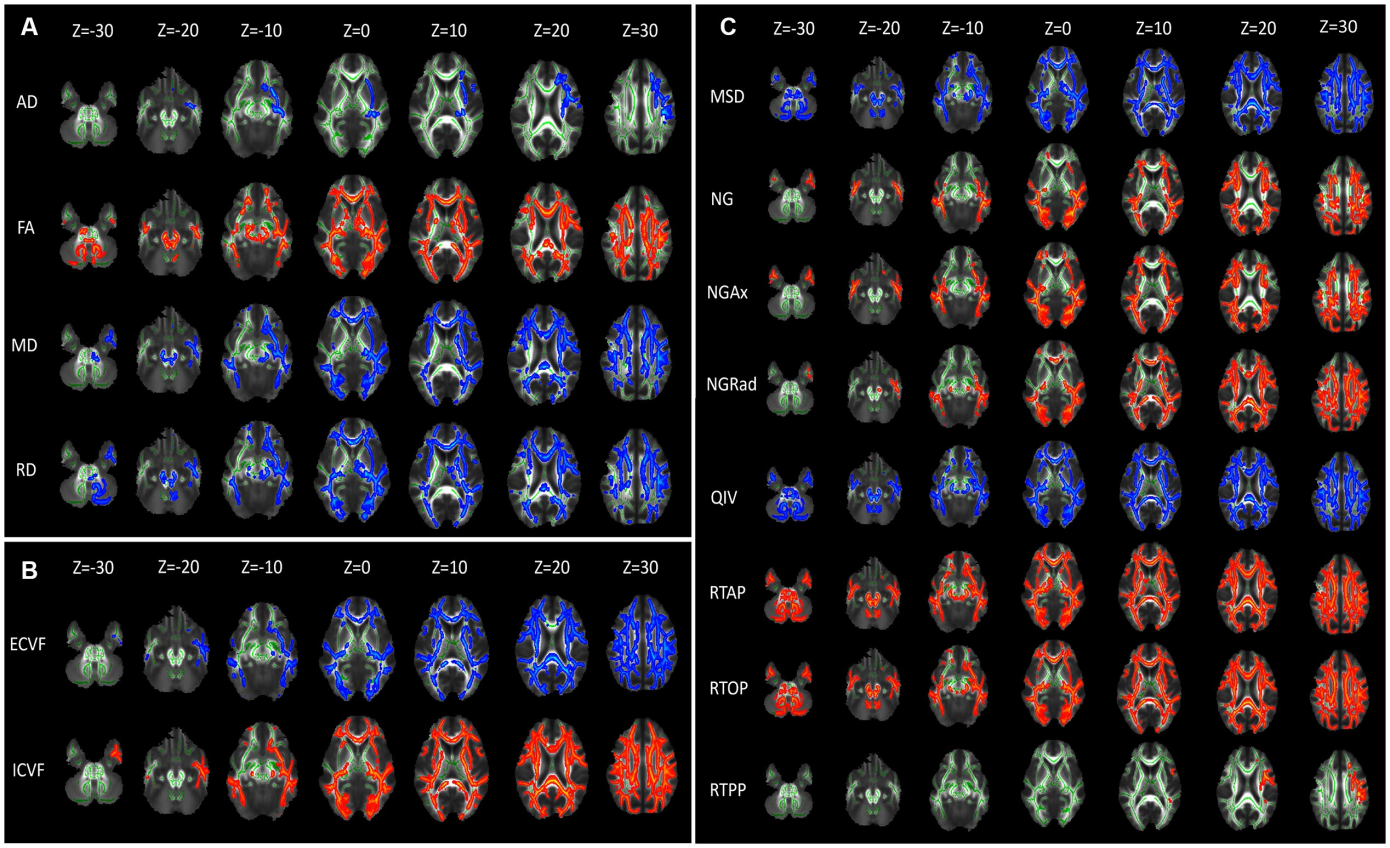
Diffusion parameter maps displaying microstructural differences between CKD patients and healthy controls. **(A)** Illustrates diffusion tensor imaging (DTI) parameters: axial diffusivity (AD), fractional anisotropy (FA), mean diffusivity (MD), and radial diffusivity (RD). **(B)** Depicts Neurite Orientation Dispersion and Density Imaging (NODDI) parameters: extracellular volume fraction (ECVF) and intracellular volume fraction (ICVF). **(C)** Shows Mean Apparent Propagator MRI (MAP-MRI) parameters: mean squared displacement (MSD), non-Gaussianity (NG), axial non-Gaussianity (NGAx), radial non-Gaussianity (NGRad), and q-space inverse variance (QIV). Each column represents axial brain slices at different Z-coordinates, indicating the spatial distribution of these parameters within the brain. Green represents the mean FA skeleton of all subjects. Red–blue represent regions with significant statistical values (*p* < 0.05).

For the DTI model, brain areas with significantly higher AD values were mainly located in the Superior corona radiata L, Superior longitudinal fasciculus L, Inferior fronto-occipital fasciculus L in the CKD group compared to the HC group (*p* < 0.001). The FA values were significantly lower in the Middle cerebellar peduncle, Genu of corpus callosum, Body of corpus callosum, Splenium of corpus callosum, Anterior corona radiata R/L, Superior corona radiata R/L, Posterior thalamic radiation R/L in the CKD group compared to the HC group. However, the MD and RD values of these brain areas were increased (*p* < 0.001; Figure 2A).

For the NODDI model, brain areas with significantly higher ECVF values were mainly located in the Genu of corpus callosum, Body of corpus callosum, Splenium of corpus callosum, and bilateral Posterior limb of internal capsule, Retrolenticular part of internal capsule, Anterior corona radiata, Superior corona radiata, Posterior corona radiata, Posterior thalamic radiation, Superior longitudinal fasciculus. The ICVF values of these brain areas were decreased (*p* < 0.001; Figure 2B).

For the MAP-MRI model, the MSD and QIV values of frontal and occipital lobes were significantly higher in the CKD group compared to the HC group (*p* < 0.001). Compared with HC group, the NG, NGAx, NGRad values of the occipital and parietal lobes were lower in the CKD group (*p* < 0.001). Meanwhile, the RTAP and RTOP values of frontal, occipital and parietal lobes were also significantly decreased in the CKD (*p* < 0.001). The RTPP values of the left Superior corona radiata and Superior longitudinal fasciculus were lower in the CKD compared to the HC group (*p* < 0.001; Figure 2C).

### 3.3 Correlation between diffusion quantitative parameters and clinical indicators

**Figure 3:**
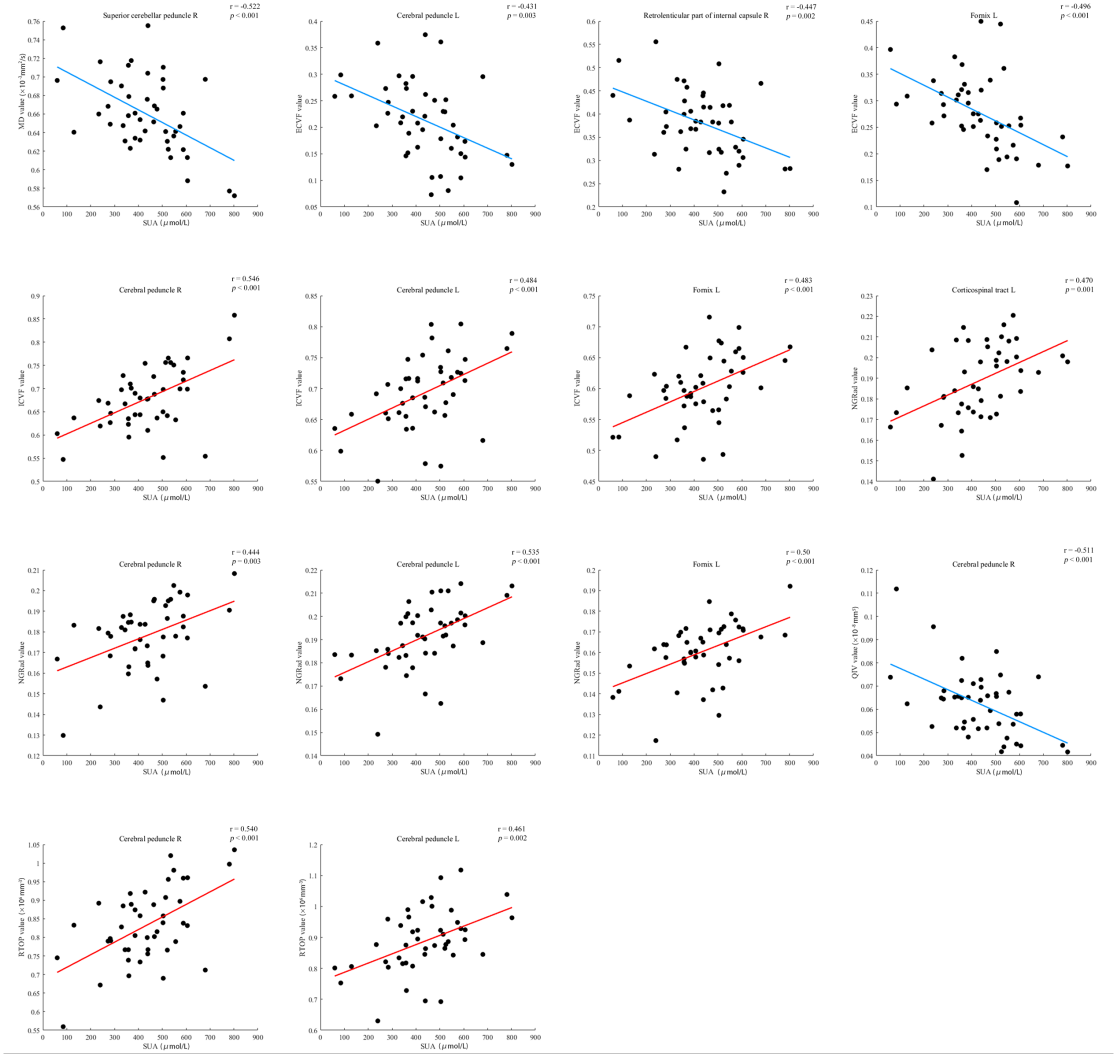
Serum uric acid (SUA) levels in CKD patients were significantly correlated with diffusion parameters in six brain regions. The regions and parameters include the corticospinal tract L, Superior cerebellar peduncle R, cerebellar peduncles R/L, Retrolenticular part of internal capsule R, and fornix L, with diffusion parameters (MD, ECVF, ICVF, NGRad, QIV, and RTOP). Each scatterplot displays the relationship between SUA levels (x-axis) and specific diffusion imaging parameters (y-axis) for the corresponding brain region, with red lines representing positive correlations and blue lines indicating negative correlations. L, left; R, right.

Associations were found between Serum Uric Acid (SUA) and diffusion parameters, including MD, ECVF, ICVF, NGRad, QIV and RTOP across multiple fiber tracts. MD values in Superior cerebellar peduncle L were negatively correlated with SUA (*r* = −0.522, *p* < 0.001). ECVF values in Cerebral peduncle L, Retrolenticular part of internal capsule R, and Fornix L were negatively correlated with SUA (*r* = −0.431 to-0.496, *p* ≤ 0.003). ICVF values in Cerebral peduncle R/L and Fornix L showed positive correlations with SUA (*r* = 0.483 to 0.546, *p* < 0.001). NGRad values in Corticospinal tract L, Cerebral peduncle R/L, and Fornix L were positively correlated with SUA (*r* = 0.444 to 0.535, *p* ≤ 0.001). QIV values in Cerebral peduncle R had a negative correlation with SUA (*r* = −0.511, *p* < 0.001). RTOP values in Cerebral peduncle R/L also correlated positively with SUA (*r* = 0.461 to 0.540, *p* ≤ 0.002; Figure 3).

### 3.4 Diagnostic performances of advanced diffusion models

**Figure 4:**
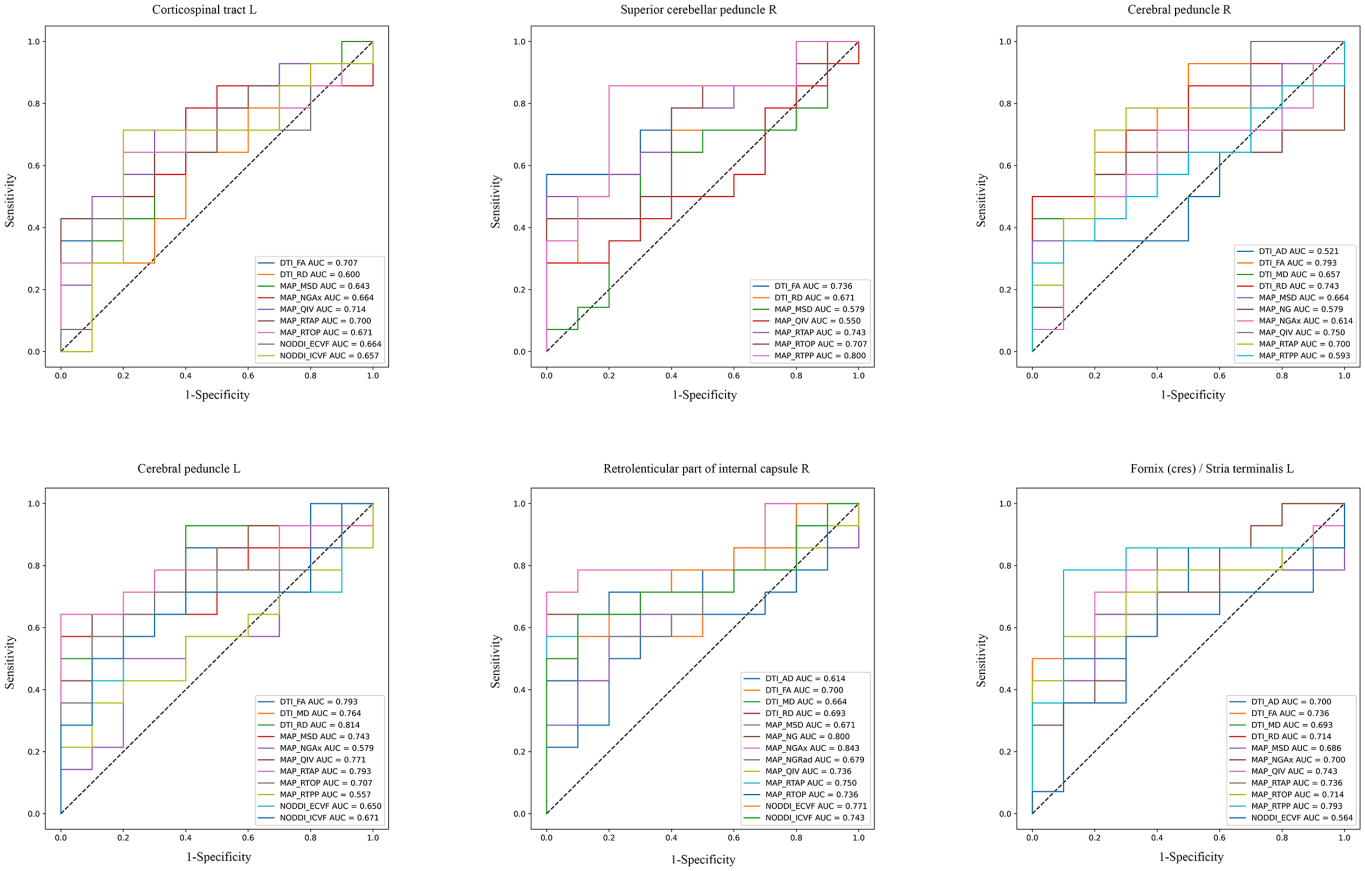
ROC curves of diffusion imaging parameters in corresponding brain regions of CKD patients. These plots compared the diagnostic capabilities of DTI, NODDI, and MAP-MRI diffusion parameters in six SUA-related brain regions (AUC > 0.5, *p* < 0.05).

ROC analysis evaluated the diagnostic capabilities of diffusion parameters in six brain regions linked to SUA, namely Corticospinal tract L, Superior cerebellar peduncle R, Cerebral peduncles R/L, Retrolenticular part of internal capsule R, and Fornix L. The AUC values varied across these regions. The DTI model’s FA parameter showed comparable performance in the above brain regions (AUC = 0.707 to 0.793, *p* < 0.05). In addition, RD of the DTI performed well in Cerebral peduncle L, with an AUC value of 0.814. In the NODDI model, the AUC values of the ECVF and ICVF of the Retrolenticular part of internal capsule R were 0.771 and 0.743, respectively (*p* < 0.05). In the MAP-MRI model, NG and NGAx in the Retrolenticular part of internal capsule R performed well (AUC = 0.80, 0.843, *p* < 0.05). MSD, QIV, and RTAP in Cerebral peduncle L, and RTOP and RTPP in Fornix L performed well (AUC = 0.714 to 0.793, *p* < 0.05; Figure 4). The results of DeLong’s tests showed that MAP_NGAx (the highest AUC value) had no statistical significance with other diffusion parameters (*p* > 0.05). Detailed DeLong’s statistical analysis results are provided in Supplementary Material 1.

## 4 Discussion

Utilizing 3 different diffusion MRI models (DTI, NODDI, MAP-MRI), our study investigated the brain microstructure in CKD patients, revealing significant differences from controls and suggesting impaired neural integrity. We found a moderate correlation between serum uric acid (SUA) and diffusion parameters in key brain areas primarily the corticospinal tract L, cerebral peduncle, retrolenticular part of internal capsule R and fornix L. These findings illuminate the complex relationship between renal function and brain structure, indicating the potential of advanced imaging in probing CKD’s neurological impacts and SUA’s role as a biomarker.

### 4.1 Diffusion quantitative parameters

For DTI, the brain of CKD patients displayed alterations in the diffusion characteristics of corpus callosum, anterior and superior corona radiata, and posterior thalamic radiation, marked by reduced FA and elevated MD and RD. In addition, we found that AD values increased in Superior corona radiata L, Superior longitudinal fasciculus L, Inferior fronto-occipital fasciculus L. The decrease of FA signifies a disruption in the microstructural integrity of the white matter. The increase in MD, which represents the mean diffusivity of water molecules, may be due to the loss of axons and myelin sheaths and the increase in extracellular fluid. Such alterations align with previous studies that reported decreased FA and increased MD in CKD patients’ white matter (8, 23, 24). The inclusion of AD and RD in our analysis offers a more comprehensive understanding, as they indicate water molecules dispersion parallel and perpendicular to the fiber orientation, hinting at axon integrity and myelin damage. Moreover, our study uniquely identified reduced FA in the middle cerebellar peduncle, with only one similar finding reported previously (24).

In our NODDI analysis, CKD patients showed higher ECVF and lower ICVF values compared to controls. The increase of ECVF is related to the diffusion of neurite blocking molecules. ICVF, correlating with neurite density (NDI) (25), decreases when nerve cells are destroyed or lost, resulting in a smaller volume of the intracellular space. In our results, NODDI detected microstructural changes in areas not identified by DTI, like the posterior limb of the internal capsule and posterior radiative crown. A study comparing DTI and NODDI to assess brain white matter abnormalities found significant differences in NDI in certain voxels not detected by DTI_FA (26). And they suggested that NODDI should be used in addition to DTI. Another previous research has also demonstrated that FA is less effective in areas with complex fiber structures (27). Obviously, our result is the support for their proposal. However, no significant changes in ODI were observed, possibly due to the limited statistical power resulting from the small sample size. This warrants further investigation through future longitudinal studies.

In complex biological tissues, water molecules’ diffusion often shows a non-Gaussian distribution due to cellular constraints (28). These microstructures cannot be adequately obtained by DTI and NODDI, but MAP-MRI can overcome this limitation. Our study revealed higher MSD and QIV values in CKD patients than in healthy individuals. MSD, is closely related to the MD metric (29, 30). But MSD is more sensitive to restricted diffusion components than MD (21, 29). The lower NG value in CKD patients, indicating neurite density, suggests reduced tissue complexity, possibly signifying axonal loss and demyelination. At present, there are limited studies on the direct relevance of MAP-MRI for chronic kidney disease. However, our findings are similar to a study on MAP-MRI in hemodialysis patients (22). Supporting this, research on Alzheimer’s disease has indicated that a decreased NG value could be indicative of axonal loss and demyelination (31). Changes in axial (NGAx) and radial (NGRad) neurite density also indicate changes in NG (11). Additionally, CKD patients had lower RTAP and RTOP values across most of the cerebrum. A previous study has reported that the family of zero displacement probabilities, including RTOP, RTAP, and RTPP, might better represent tissue microstructure changes than DTI metrics (32). The comprehensive results highlight the potential of MAP-MRI in advancing our understanding of CKD’s structural changes.

### 4.2 Correlation analysis

Our study delineates a moderate relationship between SUA levels and diffusion parameters in brain regions, particularly in the cerebral peduncle and fornix L, suggesting a link with CKD. SUA is one of the indicators of renal function. Elevated SUA correlates negatively with MD in the superior cerebellar peduncle R, hinting enhanced water molecule diffusion, a marker of diminished tissue density or microstructural impairment. Such changes are frequently linked to oxidative stress from high SUA (33, 34), potentially triggering neuronal damage and compromising brain integrity (35, 36). Furthermore, SUA demonstrated a negative correlation with ECVF in the cerebral peduncle L and fornix L, coupled with a positive correlation with ICVF. This underlines the potential role of SUA in influencing cellular stress responses or metabolic alterations. The NGRad values in the bilateral cerebral peduncles and fornix L were positively correlated with SUA. This may be due to cellular stress and metabolic changes caused by high SUA levels, leading to changes in neurons and nerve fibers, thereby increasing NGRad values. Elevated SUA also associates positively with RTOP values in these regions, implying effects on cerebral microcirculation, possibly from SUA-induced microvascular damage or hemodynamic changes (37, 38). Conversely, SUA exhibits a negative correlation with QIV in the superior cerebral peduncle R, indicating its adverse effects on vascular function, possibly reducing blood flow or causing vasoconstriction.

Overall, our results are similar to previous studies. These parallels underscore the role of elevated SUA, often resulting from impaired renal function, in inducing oxidative stress and vascular changes, thereby affecting brain structure in CKD patients. Although our results show that the correlation between SUA and brain microstructural changes in CKD patients is not obvious, our future work will address this issue by expanding the sample size.

### 4.3 Diagnostic performances of DTI, NODDI and MAP-MRI

The ROC curve analysis elucidates the diagnostic capabilities of various diffusion imaging parameters for CKD, highlighting the differential effectiveness of models in detecting CKD-related brain changes. The DTI model, particularly its FA parameter, demonstrated consistent diagnostic accuracy across multiple brain regions associated with SUA levels, with AUC values between 0.70 and 0.793. This consistency emphasizes DTI’s reliability in identifying brain microstructural alterations due to CKD, though it has limitations in capturing the full complexity of these changes compared to advanced techniques. The good diagnostic performance (0.814) of RD in Cerebral peduncle L was shown in our analysis, which can also help provide a more comprehensive understanding.

For the NOODI model, although it did not showcase outstanding AUC values in multiple brain regions, it provided critical insights into neurite density and orientation dispersion, complementing DTI and MAP-MRI results. This may be due to the small sample size of our study.

In our results, we observed that MAP-MRI’s prowess extended to multiple brain regions. In specific regions of the brain, such as the retrolenticular part of the internal capsule R, the AUC value of MAP_NGAx (0.843), was higher than other diffusion parameters, indicating potential regions where MAP-MRI may offer enhanced diagnostic sensitivity for detecting CKD microstructural changes. However, it is important to note that the DeLong’s test results did not provide robust statistical support for the overall superiority of MAP-MRI over DTI and NODDI. Therefore, while the results are promising for certain parameters in specific regions, they do not conclusively demonstrate that MAP-MRI is superior to DTI and NODDI across all regions or in a general clinical setting. But we cannot deny the ability of MAP-MRI to detect CKD-related brain changes. The advantage of MAP-MRI in predicting CKD’s brain microstructural changes needs to be verified in future studies with larger sample sizes. We will continue this work in the future to validate these preliminary findings and explore the underlying reasons for the variability in diagnostic performance between different brain regions.

These findings suggest that the three advanced diffusion MRI models could provide more detailed information in certain brain regions. Their detailed parameterization offers an additional view of CKD’s neurological changes, potentially guiding more precise and effective clinical interventions in the future.

Our study encountered several limitations. Firstly, owing to the small sample size and medication use, we found no significant differences in serum creatinine and blood urea nitrogen levels between groups. Larger future studies or those involving varied populations could further explore the relationships between these biomarkers and CKD. In addition, the cross-sectional design of our study limits causal inferences about the severity of CKD and white matter integrity. Longitudinal studies tracking the progression of CKD will enhance our understanding of its impact on white matter microstructures. Additionally, future studies requiring more refined statistical models may help elucidate the differences and potential advantages of various diffusion MRI models, such as MAP-MRI, in detecting microstructural changes associated with CKD. Moreover, future research should integrate broader clinical indicators for a more comprehensive correlation analysis with microstructural changes.

## 5 Conclusion

In conclusion, three different diffusion MRI models (DTI, NODDI, MAP-MRI) have potential as non-invasive tools for early detection of CKD-related microstructural changes. Meanwhile, further studies with larger cohorts are also necessary to validate these preliminary findings.

## Data availability statement

The raw data supporting the conclusions of this article will be made available by the authors, without undue reservation.

## Ethics statement

The studies involving humans were approved by The Ethics Committee of Zigong First People’s Hospital. The studies were conducted in accordance with the local legislation and institutional requirements. The participants provided their written informed consent to participate in this study.

## Author contributions

LH: Data curation, Investigation, Visualization, Writing – original draft. JY: Data curation, Formal analysis, Software, Validation, Writing – review & editing, Writing – original draft. CY: Investigation, Writing – review & editing. WZ: Investigation, Writing – review & editing. YH: Investigation, Writing – review & editing. LZ: Resources, Writing – review & editing. JZ: Conceptualization, Funding acquisition, Project administration, Supervision, Writing – review & editing.

## Footnotes

^1^ http://fsl.fmrib.ox.ac.uk/fsl/fslwiki/
